# Lmo1656 is a secreted virulence factor of *Listeria monocytogenes* that interacts with the sorting nexin 6–BAR complex

**DOI:** 10.1074/jbc.RA117.000365

**Published:** 2018-04-17

**Authors:** Daryl Jason David, Alessandro Pagliuso, Lilliana Radoshevich, Marie-Anne Nahori, Pascale Cossart

**Affiliations:** From the Unité des Interactions Bactéries-Cellules, Department of Cell Biology and Infection, INSERM U604, Institut National de la Recherche Agronomique USC2020, Institut Pasteur, 25 rue du Dr. Roux, 75015 Paris, France

**Keywords:** infection, host-pathogen interaction, virulence factor, sorting nexin (SNX), bacterial pathogenesis, host-pathogen interaction, Listeria monocytogenes, Lmo1656, sorting nexin 6, virulence factor

## Abstract

*Listeria monocytogenes* (*Lm*) is a facultative intracellular bacterial pathogen and the causative agent of listeriosis, a rare but fatal disease. During infection, *Lm* can traverse several physiological barriers; it can cross the intestine and placenta barrier and, in immunocompromised individuals, the blood–brain barrier. With the recent plethora of sequenced genomes available for *Lm*, it is clear that the complete repertoire of genes used by *Lm* to interact with its host remains to be fully explored. Recently, we focused on secreted *Lm* proteins because they are likely to interact with host cell components. Here, we investigated a putatively secreted protein of *Lm*, Lmo1656, that is present in most sequenced strains of *Lm* but absent in the nonpathogenic species *Listeria innocua. lmo1656* gene is predicted to encode a small, positively charged protein. We show that Lmo1656 is secreted by *Lm*. Furthermore, deletion of the *lmo1656* gene (Δ*lmo1656*) attenuates virulence in mice infected orally but not intravenously, suggesting that Lmo1656 plays a role during oral listeriosis. We identified sorting nexin 6 (SNX6), an endosomal sorting component and BAR domain–containing protein, as a host cell interactor of Lmol656. SNX6 colocalizes with WT *Lm* during the early steps of infection. This colocalization depends on Lmo1656, and RNAi of SNX6 impairs infection in infected tissue culture cells, suggesting that SNX6 is utilized by *Lm* during infection. Our results reveal that Lmo1656 is a novel secreted virulence factor of *Lm* that facilitates recruitment of a specific member of the sorting nexin family in the mammalian host.

## Introduction

The foodborne pathogen *Listeria monocytogenes* (*Lm*)^5^ can cross several physiological barriers and infect multiple cell types. The pathogenic potential of *Lm* relies on the ability of this bacterium to cross multiple physiological barriers as well as its ability to enter and replicate within a wide variety of host cell types (for recent reviews, see Refs. 1 and 2). Upon binding to host cell surface receptors, *Lm* induces its internalization into both professional phagocytes and nonphagocytic cells (for a recent review, see Ref. 2). From there, *Lm* escapes into the cytosol by rupturing its vacuole. *Lm* is able to evade host cell immune responses (for a recent review, see Ref. 3) and subvert the host cell actin cytoskeleton to drive intra- and intercellular motility (for recent reviews, see Refs. 4–6).

Secreted and surface-exposed *Lm* proteins can encounter host components and serve as virulence factors. For example, the secreted pore-forming toxin listeriolysin O (LLO) is one of the most well-characterized and potent virulence factors of *Lm* (for a review, see Ref. 7). Secretion of LLO occurs prior to *Lm* entry into the host cell. It inserts into the host plasma membrane and makes large pores. The resulting ion flux drives a diverse array of responses within the cell from global deSUMOylation (8) to mitochondrial fragmentation (9). Upon entry, *Lm* can escape into the host cytosol by lysing the phagosomal membrane through the combined actions of secreted LLO and phospholipases A and B (PlcA and PlcB) (10–12).

Recent work has uncovered novel secreted *Lm* virulence factors and their binding partners in the host cell. The secreted protein *Listeria* nuclear targeted protein A (LntA) targets the host epigenetic regulator BAHD1, altering host cell transcription (13). The small secreted protein internalin C (InlC) sequesters Tuba, a Cdc42 guanine exchange factor, to induce relaxation of membrane cortical tension, thereby facilitating increased bacterial cell-to-cell spread (14, 15). InlC also directly binds to host IκB kinase α, interfering with host innate immunity (16).

The recent plethora of genomics data and the rise of bioinformatics pipelines have enabled the rapid comparison of multiple bacterial strains and species (17–19). It is clear that the complete repertoire of proteins with which *Listeria* infects its host and targets host cell functions remains to be fully explored. Many intracellular bacteria co-opt endomembrane trafficking to promote replication and spread. The sorting nexins (SNXs) are conserved proteins that play a role in endomembrane trafficking. Their defining feature is the phox homology domain, which allows binding to different phosphoinositides (for a review, see Ref. 20). The SNX–BAR subfamily of proteins is composed of SNX1/2/5/6/32 that contain, in addition to a phox homology domain, a Bin/amphiphysin/Rvs (BAR) domain thought to sense or induce membrane curvature and tubulation as well as mediate dimerization. Heterodimers of either SNX1/2 with either SNX5/6/32 then form a complex with the core retromer components (20). The SNX–BAR–retromer complex captures endosomal cargo for retrograde trafficking to the Golgi network.

To search for novel putative virulence factors of *Listeria*, we performed a bioinformatics screen for genes present in *Lm* but absent in the closely related but nonpathogenic *Listeria innocua* (13). Here, we uncover the predicted secreted protein Lmo1656 as an additional virulence factor of *Lm*. We show that Lmo1656 is indeed a secreted protein. Lmo1656 plays a role during a murine model of infection, revealing that Lmo1656 is a *bona fide* virulence factor of *Lm*. We show that Lmo1656 contributes to virulence in mice infected via the oral but not the intravenous route, suggesting a role during the gastrointestinal phase of infection. Furthermore, we uncover the endosomal sorting protein SNX6 as a eukaryotic host target protein of secreted Lmo1656. The related sorting nexins SNX5 and SNX6 are recruited to *Lm* entry sites. Recruitment of SNX6 is abrogated when cells are infected with *Lm* Δ*lmo1656*, suggesting that Lmo1656 contributes to SNX recruitment. Other members of the SNX–BAR–retromer complex, SNX1 and SNX2, are not recruited to *Lm* entry sites, suggesting a possible differential recruitment and role of SNX–BAR proteins during infection. Together, these results uncover Lmo1656 as a secreted *Lm* virulence factor that leads to the recruitment of distinct members of the SNX–BAR–retromer complex.

## Results

### lmo1656 is conserved in Clostridia and Bacilli

**Figure 1.**
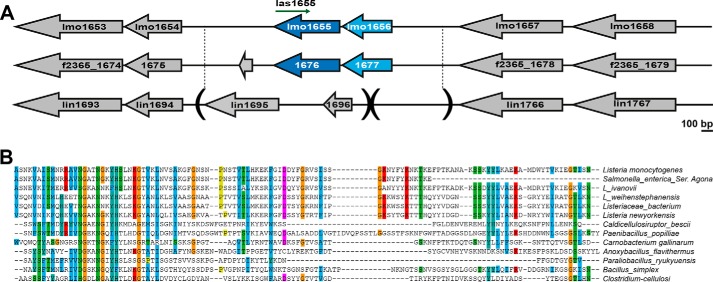
**Lmo1656 is a predicted secreted protein of *L. monocytogenes*.** *A*, synteny of the *lmo1656* locus. *lmo1656* is conserved in most sequenced strains of *L. monocytogenes* but absent in the closely related but nonpathogenic *L. innocua.* Epidemic *Lm* strain F2365 is shown as an example of a clinical isolate. *B*, homologs of *lmo1656* are predicted in other bacterial species, most of which are Gram-positive. Multiple sequence alignment (ClustalX2) of the predicted proteins, excluding the putative Sec-dependent signal peptide. The mature form of Lmo1656 is predicted to have a molecular mass of 12.49 kDa and a pI of 10.61.

To identify novel virulence factors of *Lm*, we performed a bioinformatics screen to identify putative secreted proteins that are present in *Lm* but absent in the closely related but nonpathogenic *L. innocua*. One such candidate gene, *lmo1656*, is conserved in 59 of 70 (84.3%) sequenced *Lm* strains (Fig. 1*A*) (17) and absent mainly in lineage III, which is itself poorly represented in clinical isolates. *lmo1656* is conserved in several other bacterial species, mainly the Clostridia and Bacilli classes of Gram-positive bacteria (Fig. 1*B*). Interestingly, a homolog of *lmo1656*, a *Salmonella enterica* serovar Agona hypothetical protein (NCBI Reference Sequence WP_085417617.1), is the only homolog found from a Gram-negative bacterium. However, in all cases, the function(s) of these hypothetical proteins is unknown.

The N terminus of Lmo1656 harbors a predicted signal peptide for Sec-dependent secretion. The predicted mature Lmo1656 protein is 12.5 kDa and 113 residues in length with a pI of 10.0. However, no other significant domains or motifs are apparent (SMART/Pfam). Together, these data suggest that Lmo1656 is a putative secreted, small, positively charged *Lm* candidate virulence factor.

### Lmo1656 is a secreted protein

**Figure 2.**
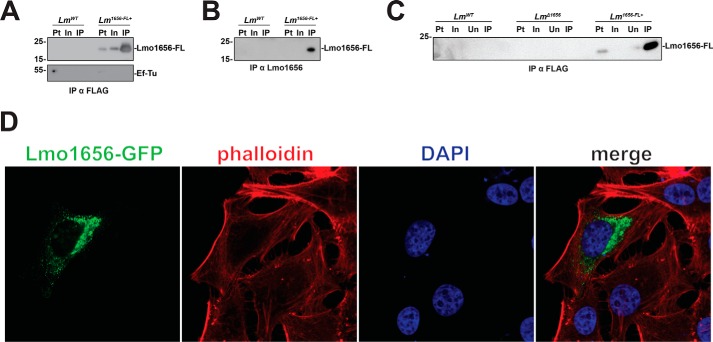
**Lmo1656 is a secreted protein.** *A*, overexpressed Lmo1656-FLAG is secreted into the growth medium. Either WT (*Lm^WT^*) or Lmo1656-FLAG-overexpressing (*Lm^1656-FL^*^+^) bacteria were grown to exponential phase in broth medium. The sterile filtered supernatant was immunoprecipitated against FLAG. Lmo1656-FLAG is immunoprecipitated in the sterile filtered supernatant of the growth medium from *Lm^1656-FL^*^+^ but not *Lm^WT^* bacteria. Data are representative of three independent experiments (*Pt*, bacterial pellet; *In*, input fraction; *IP*, immunoprecipitated fraction). *B*, secreted Lmo1656-FLAG can be immunoprecipitated by antibodies raised against Lmo1656. Either WT (*Lm^WT^*), *lmo1656* deletion mutant (*Lm*^Δ^*^1656^*), or Lmo1656-FLAG–overexpressing (*Lm^1656-FL^*^+^) bacteria were grown to exponential phase in broth medium. The sterile filtered supernatants were immunoprecipitated with a pooled mixture of affinity-purified anti-Lmo1656 polyclonal rabbit antibodies (1 μg of antibody/50 μg of protein). Samples were subjected to Western blotting with the same anti-Lmo1656 antibody pool (1:500 in TBS, Tween 20, and 5% milk) (*Pt*, bacterial pellet; *In*, input fraction; *Un*, unbound fraction postimmunoprecipitation; *IP*, immunoprecipitated fraction). *C*, overexpressed Lmo1656-FLAG is secreted into infected cells. JEG3 cells were infected (m.o.i., 20) with either WT (*Lm^WT^*) or Lmo1656-FLAG–overexpressing (*Lm^1656-FL^*^+^) bacteria. The cells were lysed 4 hpi and subjected to immunoprecipitation against FLAG. Lmo1656-FLAG is immunoprecipitated in the soluble lysate from cells infected with *Lm^1656-FL^*^+^ but not *Lm^WT^* bacteria. Data are representative of three independent experiments (*Pt*, insoluble pellet postlysis; *In*, input fraction; *IP*, immunoprecipitated fraction). *D*, overexpressed Lmo1656-GFP localizes to endomembranes. HeLa cells were transiently transfected with Lmo1656-GFP for 24 h. Actin and nuclei were stained with phalloidin and DAPI, respectively. Images were acquired with a spinning disk confocal microscope. Data are representative of at least three independent experiments.

To assess whether Lmo1656 is secreted, we assayed for its presence in growth medium supernatant. We created an *Lm* strain stably overexpressing full-length Lmo1656 tagged at the C terminus with 2xFLAG (Lmo1656-FLAG) under the constitutive pHyper promoter using the integrative plasmid pAD (21). Overexpressed Lmo1656-FLAG, but not EF-Tu (a cytosolic nonsecreted *Lm* protein used as a control) (22), is detected and can be immunoprecipitated from the growth medium supernatant of exponentially growing *Lm* using anti-FLAG resin or an antibody we generated against Lmo1656 (Fig. 2, *A* and *B*). However, we were unable to detect endogenous Lmo1656 from the bacterial pellet or the growth medium supernatant from *Lm* WT, likely due to levels of expression below the sensitivity of the antibody.

To address whether Lmo1656-FLAG can be secreted into the cytosol of infected mammalian cells, we infected human choriocarcinoma JEG3 cells with either WT or *Lm* overexpressing Lmo1656-FLAG. Using an anti-FLAG antibody, Lmo1656-FLAG can be immunoprecipitated from the soluble fraction of JEG3 cell lysate infected with *Lm* overexpressing Lmo1656-FLAG but not WT *Lm* expressing Lmo1656 at endogenous levels (Fig. 2*C*). Together, these results show that overexpressed Lmo1656 can be secreted as a soluble protein from bacteria grown in brain–heart infusion growth medium (BHI) and from infected mammalian cells.

We next sought to examine the localization of Lmo1656 in the cytoplasm of host cells. We transiently transfected HeLa cells with plasmids encoding Lmo1656-GFP and analyzed its localization by confocal microscopy. Notably, overexpressed Lmo1656-GFP localized to puncta scattered throughout the cytoplasm with enrichment around the perinuclear region (Fig. 2*D*). This pattern is reminiscent of proteins involved in endomembrane trafficking, suggesting that Lmo1656 might regulate host membrane trafficking during *Listeria* infection. Altogether, our results demonstrate that Lmo1656 can be secreted from bacteria and localizes to endomembranes.

### Lmo1656 is a virulence factor

**Figure 3.**
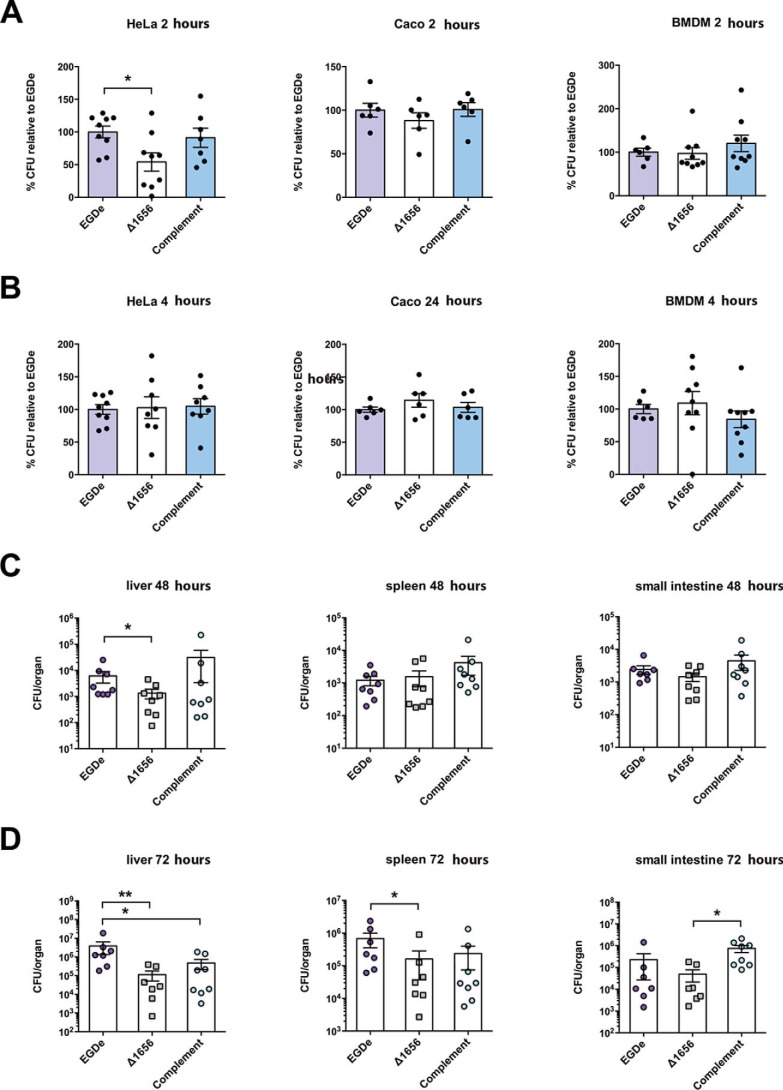
**Lmo1656 is a *bona fide* virulence factor of *L. monocytogenes*.** *A* and *B*, Lmo1656 contributes to early infection in certain cell types. *Lm*^Δ^*^lmo1656^* have decreased bacteria at early time points (*A*; *t* = 2 hpi) but not later (*B*; *t* = 4 or 24 hpi) of infection in HeLa but not Caco2 or mouse bone marrow–derived macrophages (*BMDM*). Results are normalized to the mean cfu for WT per replicate (*n* = 3 wells per replicate over at least two independent replicates; *, *p* = 0.0339, analysis of variance). *C* and *D*, Lmo1656 contributes to oral infection *in vivo*. Knockin mice expressing a “humanized” E-Cad^E16P^ were infected with *Lm^WT^*, *Lm*^Δ^*^lmo1656^*, or the complemented *Lm*^Δ^*^lmo1656^*^+^*^C^* via oral gavage. Mouse livers have decreased bacterial burden both 48 (*C*) and 72 hpi (*D*) when infected by *Lm*^Δ^*^lmo1656^*, whereas spleens have decreased bacterial burden 72 hpi (*D*) (*n* = 7–8 mice per *Lm* genotype; *, *p* = 0.0261 Mann–Whitney *U* test; data are means ± S.D. of at least three independent experiments).

To test whether Lmo1656 plays a role during infection, we infected tissue culture cells and mice. We first complemented the Δ*lmo1656* mutant by chromosome integration of a plasmid encoding the entire *lmo1656* ORF preceded by the putative *lmo1656* promoter region. We then infected tissue culture cells with the *Lm* WT, deletion mutant, and its complemented strain and assayed for recovered bacteria surviving the standard gentamicin assay for internalized bacteria. We found that there were fewer *Lm* Δ*lmo1656* at early time points of infection in HeLa cells as compared with infection with *Lm* WT. In contrast, there was no difference in bacterial counts between *Lm* WT and Δ*lmo1656* in Caco2 cells and bone marrow–derived macrophages (Fig. 3, *A* and *B*). Because entry into HeLa cells is mainly dependent on the host receptor Met, whereas entry into the other assayed cell types can utilize both the Met receptor and E-cadherin (2), our results suggest that Lmo1656 may play a role during early Met-dependent infection. Lmo1656 could potentially promote adherence to cells and/or endocytosis of *Lm* or escape from the primary vacuole, two early steps in *Lm* infection.

To assess whether Lmo1656 is a virulence factor *in vivo*, we infected mice with *Lm* WT, Δ*lmo1656*, and its complemented strain. Deletion of *lmo1656* had no effect on bacterial burden in the liver or spleen 72 h postinfection (hpi) in intravenously infected BALB/c mice (Fig. S1A). To assess whether *lmo1656* contributes to oral listeriosis, we infected mice that express humanized E-cadherin^E16P^ via oral gavage. This point mutation in E-Cad mimics the docking site of human E-Cad with the *Lm* surface protein internalin A and renders mice more susceptible to *Lm* oral infection (23). Whereas intravenously infected mice displayed no difference in infection, orally inoculated mice infected with Δ*lmo1656* had a reduction in bacterial burden in the liver 48 and 72 hpi (Fig. 3, *C* and *D*). Notably, there is also a decrease 72 hpi in spleens of mice infected with *Lm* Δ*lmo1656*. There is no difference in bacterial burden in the mesenteric lymph nodes or the intestinal content among the three *Lm* strains either at 48 or 72 hpi (Fig. S1B). Although we see no difference in intracellular *Lm* WT and *Lm* Δ*lmo1656* in the small intestine, there is a significant difference between *Lm* Δ*lmo1656* compared with its complemented strain (Fig. 3*D*). Therefore, Lmo1656 contributes to *Lm* virulence during oral, but not intravenous, infection *in vivo*.

### Listeria entry recruits distinct members of the SNX–BAR family

**Figure 4.**
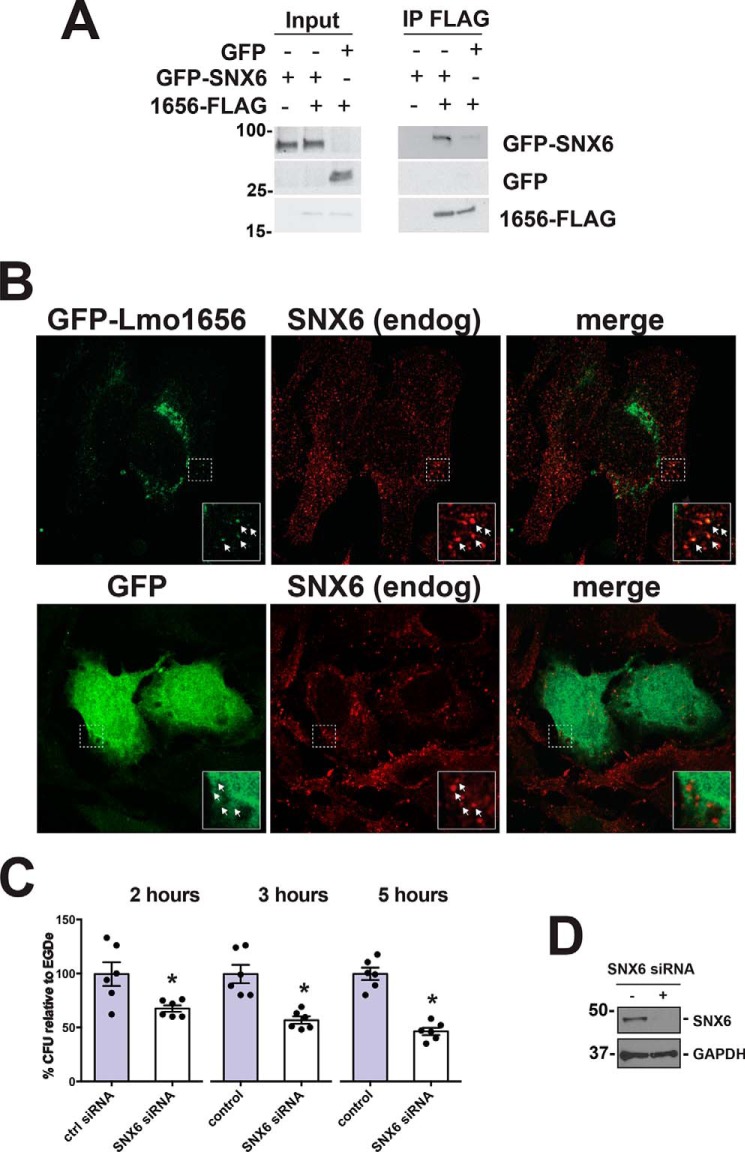
**SNX6 and Lmo1656 interact biochemically and genetically.** *A*, GFP-SNX6 and Lmo1656-FLAG biochemically interact. HeLa cells were transiently transfected with GFP-SNX6 or GFP and either empty vector (−) or Lmo1656-FLAG (+). Anti-FLAG immunoprecipitation (*IP*) was performed on clarified lysate 48 h post-transfection. GFP-SNX6 can be coimmunoprecipitated with Lmo1656-FLAG from cells transfected with Lmo1656-FLAG but not with empty vector. Results are representative of two independent experiments. *B*, Lmo1656-GFP colocalizes with SNX6. HeLa cells were transiently transfected with Lmo1656-GFP (*upper panel*) or GFP (*lower panel*) and stained for endogenous (*endog*) SNX6. *Inset*, magnification of colocalizing Lmo1656-GFP and SNX6. *C*, SNX6 contributes to *Lm* infection. HeLa cells were transiently transfected with either nontargeting siRNA pool (control (*ctrl*)) or an siRNA pool targeting SNX6. 72 h post-transfection, cells were infected with *Lm* EGDe-PrfA* and lysed 2, 3, and 5 hpi. (*, *p* < 0.05; data are means ± S.D. of at least three independent experiments). Results are in triplicate from two independent experiments. *D*, knockdown of SNX6 in HeLa cells. HeLa cells were either treated with scrambled siRNA (−) or siRNA against SNX6 (+). After 72h, protein levels were analyzed by Western blotting with the indicated antibodies.

To assess the mechanisms by which Lmo1656 contributes to virulence, we sought possible eukaryotic binding proteins of Lmo1656. Using a human placental cDNA library as bait, we performed a yeast two-hybrid screen against the predicted mature form of Lmo1656. We identified the sorting nexin BAR protein SNX6 as a predicted high-confidence direct interaction partner of Lmo1656. To test whether Lmo1656 and SNX6 do interact, we transiently transfected HeLa cells with plasmids encoding GFP-SNX6 and plasmids encoding either Lmo1656-FLAG, empty vector, or GFP alone. With an anti-FLAG immunoprecipitation, we were able to coimmunoprecipitate GFP-SNX6 with Lmo1656-FLAG (Fig. 4*A*). These results indicate that Lmo1656 and SNX6 can biochemically interact.

Immunofluorescence analysis using Lmo1656-GFP showed colocalization of Lmo1656 and endogenous SNX6 in a subset of vesicular structures scattered throughout the cytoplasm (Fig. 4*B*). However, when Lmo1656 was tagged with FLAG, its localization appeared more diffuse (Fig. S2). To ensure that the vesicular localization of Lmo1656-GFP was not due to the GFP, we expressed GFP alone. GFP showed a diffuse staining throughout the cytoplasm and did not colocalize with endogenous SNX6 (Fig. 4*B*). It is possible that the positive charge of the FLAG tag at least partially altered Lmo1656 localization.

**Figure 5.**
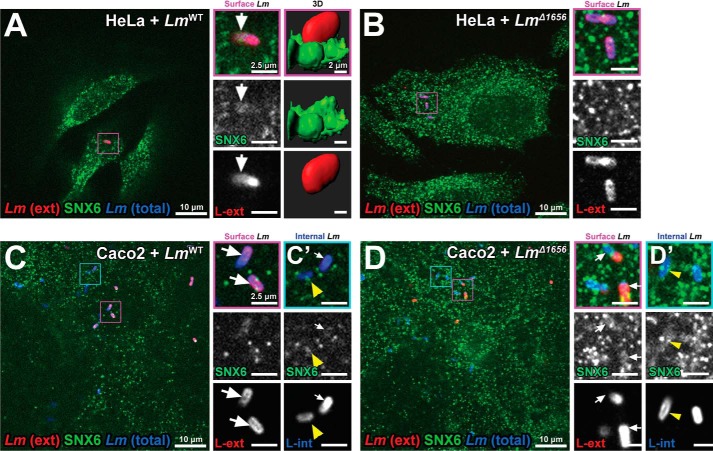
**SNX6 colocalizes with *Lm* entry sites in an Lmo1656-dependent manner.** *A* and *B*, endogenous SNX6 is recruited to internalizing *Lm* in an Lmo1656-dependent manner in HeLa cells. HeLa cells infected with either *Lm^WT^* (*A*) or *Lm*^Δ^*^1656^* (*B*) (m.o.i., 40; 2 hpi) and stained for external (*ext*) and internalized bacteria. *A*, *right*, 3D surface reconstruction of endogenous SNX6 recruited to *Lm^WT^* entry sites (Imaris). *C* and *D*, endogenous SNX6 is recruited to internalizing *Lm* in an Lmo1656-independent manner in Caco2 cells. Caco2 cells were infected with either *Lm^WT^* (*C*) or *Lm*^Δ^*^1656^* (*D*) (m.o.i., 10; 2 hpi) and stained for external bacteria. Internalized (*int*) *Lm^WT^* (*C*′) or *Lm*^Δ^*^1656^* (*D*′) *Lm* colocalize with SNX6. Magnification of bacteria (*purple squares*) is shown in adjacent images. Data are representative of at least three independent experiments; controls (*Lm^WT^* or *Lm*^Δ^*^1656^*) for each cell type were imaged with spinning disk confocal microscope during the same session using identical settings and, where necessary, identical adjustments for brightness and contrast. (*A* and *B versus C* and *D*).

We subsequently tested whether SNX6 contributes to *Lm* infection. We had previously performed a genome-wide RNAi screen and found SNX6 as one of the top candidate genes contributing to *Lm* infection (24). However, the bacterial strain *Lm* EGDe PrfA*, which is more invasive than the reference strain *Lm* EGDe, was used in this screen. When we used *Lm* EGDe PrfA*, we independently confirmed these results (Fig. 4, *C* and *D*). We then sought to determine the role of SNX6 during infection. Because Lmo1656 contributes to early infection of certain types of cultured cells (Fig. 3, *A* and *B*), we first assessed whether the subcellular localization of its putative host target, SNX6, is affected during infection. Using differentially labeled surface-exposed *versus* internalized *Lm* (25), we found that endogenous SNX6 was recruited to invading *Lm* in HeLa and Caco2 cells (Fig. 5, *A* and *C*). We then assessed whether Lmo1656 contributes to the recruitment of SNX6 to internalizing *Lm*. Endogenous SNX6 colocalizes with surface-localized and some internalized *Lm* WT but not with *Lm* Δ*lmo1656* in HeLa cells (Fig. 5, *A* and *B*). Colocalization analysis revealed that 93% of extracellular *Lm* colocalize with SNX6, whereas only 7% of extracellular *Lm* Δ*lmo1656* colocalize with SNX6. However, no such difference is convincingly displayed when infecting Caco2 cells (Fig. 5, *C* and *D*). In addition, we found that SNX6 transiently associates with invading *Lm* as the colocalization between *Lm* and SNX6 drops to 40% once bacteria are internalized. These results suggest that SNX6 is transiently recruited to internalizing *Lm* in an Lmo1656-dependent manner in cell types that utilize InlB/Met entry, a finding that parallels the effect of Lmo1656 on early steps of infection (Fig. 3, *A* and *B*).

**Figure 6.**
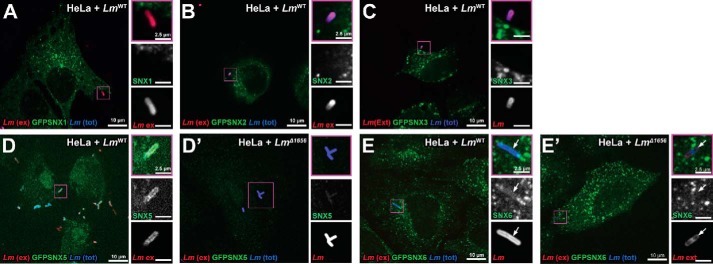
**Certain GFP-SNX–BAR proteins are recruited to *Lm* entry sites in an Lmo1656-dependent manner.** *A–C*, *Lm* entry sites do not recruit a subset of GFP-SNX proteins. HeLa cells were transiently transfected with GFP-SNX constructs and stained for external (*ex*) *Lm* (m.o.i., 20; 2 hpi). *A*, GFP-SNX1; *B*, GFP-SNX2; *C*, GFP-SNX3. *D* and *E*, *Lm* entry sites recruit some GFP-SNX–BAR family proteins. HeLa cells were transiently transfected with GFP-SNX–BAR constructs and stained for external (*ext*) *Lm^WT^. D* and *D*′, GFP-SNX5 with *Lm^WT^* or *Lm*^Δ^*^1656^*, respectively; *E* and *E*′, GFP-SNX6 with *Lm^WT^* or *Lm*^Δ^*^1656^*, respectively. Magnification of bacteria (*purple squares*) is shown in adjacent images. Data are representative of at least three independent experiments; controls (*Lm^WT^* or *Lm*^Δ^*^1656^*) were imaged with a spinning disk confocal microscope during the same session using identical settings and, where necessary, identical adjustments for brightness and contrast. *tot*, total.

Because the SNX–BAR proteins assemble as a heterodimer of either SNX1/2 with SNX5/6 as part of the SNX–BAR–retromer complex (20), we wondered whether other components of the SNX–BAR complex are also recruited to internalizing *Lm*. We transiently transfected HeLa cells with GFP-SNX constructs (26, 27) to assess their subcellular localization upon infection. GFP-SNX6 colocalizes in HeLa cells with WT adherent and internalizing *Lm*, but not with *Lm* Δ*lmo1656* (Fig. 6, *E* and *E*′), similarly to endogenous SNX6 (Fig. 6*A*). GFP-SNX5 (Fig. 6, *D* and *D*′) is also recruited to *Lm* entry sites in an Lmo1656-dependent manner. We found that 91.5% of *Lm* colocalized with SNX5 at 2 hpi. Interestingly, the association of SNX5 with *Lm* seems to have different dynamics compared with SNX6. Indeed, SNX5 colocalized with internalized *Lm* to a greater extent (82%) compared with SNX6 (40%), suggesting a differential role of the two proteins during infection. In contrast, neither GFP-SNX1 nor GFP-SNX2 is recruited to internalizing *Lm* (Fig. 6, *A* and *B*). This is somewhat surprising because either SNX1 or SNX2 is known to form a heterodimer with either SNX5 or SNX6 (20, 28). Additionally, an unrelated sorting nexin, GFP-SNX3 (Fig. 6*C*), is not recruited to *Lm* entry sites, suggesting a specific recruitment of SNX5/6 to internalizing *Lm*.

Together, our results have uncovered Lmo1656 as a novel secreted virulence factor in *Lm* infection. They suggest a role of Lmo1656 in recruiting some members of the SNX–BAR subfamily of proteins to *Lm* entry sites.

## Discussion

In this report, we describe the secreted protein Lmo1656 as a novel virulence factor during *L. monocytogenes* infection. This small positively charged protein is secreted from bacteria and interacts with the SNX–BAR subfamily protein SNX6. Deletion of *lmo1656* lowers infection levels in certain cell types during early infection *in vitro* and reduces bacterial load in the liver following oral infection *in vivo*. Certain members of the SNX–BAR subfamily proteins are recruited to sites of *Lm* entry, suggesting that *Lm* infection may assemble an infection-specific sorting nexin complex. In addition, SNX6 is required for effective *Lm* infection.

We used comparative genomics to identify putative virulence factors absent in nonpathogenic *Listeria* species but present in *Lm*. Our data revealed Lmo1656 as a *bona fide Lm* virulence factor. Homologs of *lmo1656* are found in most sequenced strains of *Lm* but are mainly absent in *Lm* lineage III strains, which are poorly represented in clinical isolates (29, 30). Strikingly, we found homologs of *lmo1656* in other pathogenic bacterial species, mainly in the Gram-positive Bacilli and Clostridia. One notable exception is the *lmo1656* homolog in the Gram-negative *S. enterica* subsp. *enterica* serovar Agona, which has recently been implicated in human disease (31–33). This homolog in *Salmonella*, although sharing a high degree of identity with *lmo1656*, is predicted to encode a smaller protein and could thus interact with different cellular partners than Lmo1656. Whether Lmo1656 homologs have conserved functions in these other species remains to be explored.

We found Lmo1656 to play a role during an early step of infection in HeLa cells, a cell line that relies on Met receptor for *Lm* invasion. Because deletion of *lmo1656* lowers bacterial load in HeLa cells at early time points, this suggests that Lmo1656 might be implicated either in the interaction of *Listeria* with target cells (by modulating adhesion and/or internalization) or in the escape from the primary vacuole via a still unidentified mechanism. *Lm* entry is known to recruit, in addition to Met, a number of host cell components, including EEA1, Cbl, clathrin, clathrin adaptor protein-1, and dynamin (34, 35), and was recently found to recruit components of the exocytic machinery (36). We have uncovered that certain members of the SNX–BAR subfamily of proteins are recruited to sites of *Lm* entry. Because the SNX–BAR complex is thought to be composed of a heterodimer of SNX1/2 and SNX5/6 (20), differential recruitment could constitute novel infection-related SNX–BAR complexes (24). We determined that Lmo1656 interacts with SNX6, which we had previously identified as a human gene modulator of *Lm* infection through a genome-wide RNAi screen (24). Notably, SNX6 is the only member of the SNX–BAR subfamily that significantly contributes to *Lm* infection of HeLa cells as the results from the genome-wide screen for the other components of the SNX–BAR–retromer complex were less clear. Interestingly, the distinct effects of different sorting nexins on infection parallel the noncanonical recruitment of SNX5/6 to sites of *Lm* entry. Recently, an unrelated sorting nexin, SNX10, was implicated in controlling *Lm* infection in mouse macrophages (37) through an effect on phagosomal maturation. In contrast, SNX6 is required for productive infection in epithelial cells, and *lmo1656* deletion does not alter bacterial growth in macrophages. It is tempting to speculate that distinct sorting nexins, which have different roles during endomembrane trafficking, have distinct effects during bacterial infection. We had initially hypothesized that Lmo1656 could disrupt the retromer complex, but because we did not find evidence to support that hypothesis, we cannot exclude that sorting nexins 5 and 6 could in some way act as sensors of *Listeria* infection. Future studies will elucidate which of these potential mechanisms is at play during *Listeria* infection.

Interestingly, SNX–BAR proteins are recruited to *Chlamydia trachomatis* inclusion bodies (38) via direct interaction with a secreted virulence factor (38–41). In this case, all members of the SNX–BAR family (SNX1/2/5/6) are recruited to the inclusion bodies and induce membrane tubulation. SNX–BAR proteins then reduce *Chlamydia* infectivity (38), possibly by promoting lysosomal function, although this is currently unclear (40). Here, we show an interaction between a secreted *Lm* protein and the sorting nexin-BAR family proteins. Notably, only a distinct subfamily of the SNX–BAR proteins appears to be recruited by *Lm* to sites of invasion. It will be interesting to test whether *Lm* perturbs, in addition to lysosomal integrity (42), lysosomal trafficking during early infection.

Sorting nexins are also targeted by *S. enterica* (for a recent review, see Ref. 43). SNX1 is recruited during early *Salmonella* infection to *Salmonella*-containing vacuoles (SCVs) where SNX1 facilitates the removal of CI-MPR from SCVs (44). SNX3 is also recruited to SCVs and plays a role in the recruitment of host factors and thus a role in SCV maturation (45) Other work has further implicated the disruption of CI-MPR trafficking (46). A recent proteomics approach analyzing SCVs has also revealed the core retromer component VPS26B as a member of SCVs (47). It would be appealing to determine whether the homolog of Lmo1656 in *Salmonella* plays a role in SNX1 or SNX3 recruitment during infection.

Notably, Lmo1656 plays a role during infection *in vivo*. The lack of a detectable effect in intravenously infected mice compared with orally infected mice is intriguing. The observation that after oral infection there is no difference in bacterial load in intestinal content or in intestinal cells despite a significant difference in bacterial load in the liver strongly suggests that Lmo1656 positively affects transcytosis in the intestine across the goblet cells. We propose that transcytosis of bacteria may be controlled by an Lmo1656-dependent effect on SNX proteins.

## Experimental procedures

### Molecular cloning

Deletion of *lmo1656* was performed as described previously (13). Briefly, PCR products of ∼600 bp upstream and downstream of the *lmo1656* open reading frame (ORF) were fused via splicing by overlap extension into the pMAD vector with appropriate restriction sites. To create a plasmid for complementation of deletion mutants, a pAD plasmid (21) was created with the full-length *lmo1656* ORF with the predicted promotor (200 bp upstream of the detected transcription start site) to generate the plasmid pEndo-1656. To create a plasmid for the overexpression of full-length Lmo1656-FLAG, the entire *lmo1656* ORF with a 2xFLAG at the C terminus was synthesized as a gBlock (Integrated DNA Technologies) and subcloned into the integrative plasmid pAD (21) using the appropriate restriction sites.

To create a plasmid for the overexpression of Lmo1656-FLAG in mammalian cells, the cDNA encoding the predicted mature form of Lmo1656 was codon-optimized for human expression and synthesized (GeneCust) with 2xFLAG at either the N or C terminus of Lmo1656. The resulting construct was then subcloned into pcDNA3.1 using the appropriate restriction sites. To create a plasmid for the overexpression of Lmo1656-GFP in mammalian cells, the codon-optimized form of *lmo1656* was amplified by PCR and subcloned into pEGFP-C2 (Clontech) using the appropriate restriction enzymes.

### L. monocytogenes mutant construction

Electrocompetent *Lm* were transformed using standard methods (48). Briefly, a culture of *Lm* was grown overnight in BHI. This overnight culture was diluted in fresh BHI supplemented with 500 mm sucrose (sterile filtered), then later supplemented with 10 μg/ml ampicillin, and grown shaking at 37 °C to exponential phase (*A*_600 nm_ = 0.8–1). The bacterial pellet was washed several times in cold electroporation buffer (10% glycerol, 500 mm sucrose, pH 7, sterile filtered) and then snap frozen in aliquots. *Listeria* was electroporated with 1 μg of plasmid. To verify the lack of off-target mutations, the chromosomes of EGDe and two independent strains of EGDe-Δ*lmo1656* were sequenced (Genopole, Institut Pasteur).

### Generation of antibodies

Peptide fragments encoding 17 residues near the N terminus and C terminus of Lmo1656 were generated (RRAVNGATNGKYHSLNK and EKAMDWYTVKIEGTISN, respectively) and coupled to keyhole limpet hemocyanin to create antigens (GeneCust). Two separate rabbits were injected with each antigen supplemented with Freund's adjuvant (Covalabs). Each of the resulting affinity-purified antibodies was then pooled separately and dialyzed to 1 mg/ml in phosphate-buffered saline (PBS) and 50% glycerol.

### Secretion assays

*Lm* overexpressing the full-length Lmo1656 and tagged at the C terminus with 2xFLAG were grown in BHI to exponential phase. After centrifugation, the BHI supernatant was kept on ice and then sterile filtered (0.2 μm). ∼2 μg of protein (Bradford assay) were subjected to immunoprecipitation overnight at 4 °C with either 20 μl of wet packed beads of M2-agarose (Sigma) or rabbit antibodies raised against Lmo1656 (0.5 μg of affinity-purified antibody) followed by precipitation with Protein A-agarose beads (GE Healthcare).

For intracellular secretion assays, exponentially growing *Lm* were washed three times in PBS and then used to infect JEG3 cells at a multiplicity of infection (m.o.i.) of 20 for a total of 4 hpi. Cells were lysed in 50 mm Tris-HCl, pH 8.0, 150 mm NaCl, 1 mm EDTA, 1% Triton X-100, phosSTOP (Roche Applied Science), and Complete protease inhibitor mixture (Roche Applied Science). The crude cell lysate was centrifuged at 5000 × g for 15 min at 4 °C to remove cellular debris, nuclei, and bacteria. ∼2 μg of protein from the soluble cell fraction were subjected to immunoprecipitation overnight at 4 °C with M2-agarose beads (Sigma).

### Animal infections

Animal experiments conformed to the Council Directive of November 24, 1986 on the approximation of laws, regulations, and administrative provisions of the member states regarding the protection of animals used for experimental and other scientific purposes (86/609/Eec). Experiments that relied on laboratory animals were performed in strict accordance with the Institut Pasteur's regulations for animal care and use protocol, which was approved by the Animal Experiment Committee of the Institut Pasteur (approval number 03-49).

Inocula for intravenous and oral infections were prepared as described previously (49). Briefly, an overnight *Lm* culture was diluted in fresh BHI and grown with shaking at 37 °C until an *A*_600 nm_ of 0.8–1. Bacteria were centrifuged several times and washed with cold saline, resuspended with saline, and then snap frozen into aliquots using liquid nitrogen.

For intravenous infections, 8–10-week-old BALB/c female mice were inoculated with 10^4^ cfu and dissected 72 hpi. For oral infections, BALB/c were gavaged with 5 × 10^9^ cfu in saline supplemented with PBS/CaCO_3_. Transgenic mice expressing E-Cad^E16P^ (50) were orally infected by gavage with 10^9^ cfu in saline supplemented with PBS/CaCO_3_ and dissected 24, 48, and 72 hpi. For all infections, inocula were plated to control for the number of *Lm*. Small intestines were washed five times in DMEM, incubated in DMEM with 100 μg/ml gentamicin for 2 h at room temperature, and then washed again five times in DMEM prior to sonication (49). Serial dilutions of organ homogenates were plated onto either BHI-agar plates (pancreas and liver), BHI-agar plates supplemented with 50 μg/ml nalidixic acid (mesenteric lymph nodes and small intestines), or selective Oxford plates (intestinal contents).

### Cell culture and infection

Cells were grown in appropriate medium. HeLa and JEG3 cells were transiently transfected with Lipofectamine 2000 (plasmids; Invitrogen) or Lipofectamine RNAiMAX (RNAi; Invitrogen) using the manufacturer's instructions.

Infection of cultured cells was performed as described previously (25). Briefly, exponentially growing *Lm* strains (Table 1) were washed three times with PBS and used to infect HeLa (m.o.i., 40), JEG3 (m.o.i., 10), Caco2 (m.o.i., 10), and bone marrow–derived macrophages (m.o.i., 5). After 1 h of infection in minimum Eagle's medium, cells were washed one time with complete cell culture medium supplemented with 50 μg/ml gentamicin and then incubated with complete cell culture medium supplemented with 50 μg/ml gentamicin. At the desired time points, cells were washed two times with minimum Eagle's medium and then lysed with 0.1% Triton X-100. Cell lysates were serially diluted in saline and then spread onto BHI-agar plates. cfus were counted either manually or with an automatic colony counter (Scan 500, Interscience).

**Table 1:** Bacterial lines used

Name	Characteristics	BUG	Ref.
pMAD-Δ*lmo1656*	*E. coli* with plasmid bearing 600 bp upstream and downstream of *lmo1656* ORF	BUG3607	This work
pCDNA-1656^mat.codopt-^Ct2F	*E. coli* with plasmid encoding for C-terminal 2xFLAG-tagged *lmo1656*, codon-optimized for mammalian expression in the pCDNA3.1 plasmid	BUG3930	This work
pEndo-1656	*E. coli* with pAD plasmid encoding for full-length *lmo1656* under the control of the putative endogenous *lmo1656* promoter (236 bp upstream of predicted translation initiation site of *lmo1656*)	BUG3734	This work
pAD-pHyper1656Ct2F	*E. coli* with pAD plasmid encoding for full-length *lmo1656* tagged with 2xFLAG at the C terminus under the control of the pHyper promoter	BUG4198	This work
*L. monocytogenes* EGDe	WT reference strain	BUG1600	51
EGDe Δ*lmo1656*	EGDe with removal of the complete *lmo1656* ORF, clone 3-3	BUG3698	This work
EGDe Δ*lmo1656* + pEndo1656	EGDe with removal of the complete *lmo1656* ORF, complemented with the integrative plasmid pEndo-1656 encoding *lmo1656* under its putative endogenous promoter	BUG3740	This work
EGDe + pHyper 1656-Ct2xFLAG	EGDe with an integrated plasmid for overexpression of full-length *lmo1656* tagged at the C terminus with 2xFLAG	BUG4199	This work
EGDe PrfA*	EGDe with a constitutively active form of PrfA (positive regulatory factor A)	BUG3057	19
EGDe PrfA* Δ*hly*Δ*plcA*Δ*plcB*	EGDe with a constitutively active form of PrfA (positive regulatory factor A) and triple mutant of *hly* and phospholipases A/B	BUG3648	52

### Identification of binding partners of Lmo1656

A yeast two-hybrid screen was performed (Hybrigenics) using the predicted mature form of Lmo1656 (amino acids 31–143; fusion N-LexA-Lmo1656-C) as the bait with a human placental cDNA as the source of prey. After screening 56.8 million interactions, two proteins were identified as “very high confidence” interactors of Lmo1656: SIPA1L1 (GenBank^TM^ accession number AF090990.1) and SNX6 (GenBank accession number NM_152233.2).

### Immunofluorescence preparation and analysis

#### Sample preparation

Samples were prepared as described previously (25). Briefly, tissue culture cells on glass coverslips were fixed in PBS and 4% paraformaldehyde for 20 min, washed twice in PBS, and washed twice in PBS and 1% BSA. Samples were stored in PBS, 1% BSA, and 0.03% sodium azide at 4 °C for at least overnight until further processing.

Antibodies at the appropriate dilutions (Table 2) in PBS and 1% BSA (surface staining) or PBS, 1% BSA, and 0.1% Triton X-100 (permeabilized/total staining) were incubated with fixed samples for 20 min at room temperature; washed three times in PBS and 1% BSA; and then incubated with the appropriate Alexa Fluor–conjugated goat secondary antibodies (Sigma). Cells were permeabilized with PBS, 1% BSA, and 0.1% Triton X-100 when required. Samples were mounted in Aqua-Poly/Mount (Tebu-Bio) and allowed to clear for at least overnight at 4 °C.

**Table 2:** Antibodies for immunofluorescence and Western blotting IF, immunofluorescence; WB, Western blotting.

Antigen	Antibody	Dilution (IF/WB)
Whole *L. monocytogenes*	R11 rabbit polyclonal	1:1000 (IF)
Human SNX6	S6324 mouse monoclonal	1:500 (IF)/1:1000 (WB)
Lmo1656 (C terminus)	R232 + R233 rabbit polyclonal	1:100 (5 μg/ml, IF)/1:500 (1 μg/ml, WB)
Actin	Phalloidin-Alexa Fluor 546	1:1000 (IF)
FLAG epitope	M2 mouse monoclonal	1:1000 (IF)/1:2000 (WB)
*Lm* EF-Tu	R114 rabbit polyclonal	1:5000 (WB)

#### Image acquisition and analysis

Confocal z-stacks (0.3-μm step size) were acquired on a Zeiss AxioObserver.Z1 inverted fluorescence microscope equipped with an Evolve electron-multiplying charge-coupled device camera (Photometrics) and a Yokogawa CSU-X1 spinning disk confocal system. Images were acquired using MetaMorph with a 100× oil objective with a numerical aperture of 1.4.

Images were visualized using Icy software (51). When necessary, comparisons of experiments and controls were acquired in the same session with the same image acquisition parameters and data analysis. 3D surface reconstructions of *Lm* with SNX6 were assembled using Imaris (Bitplane) using identical processing and thresholding when appropriate.

For the figures, images were assembled using Photoshop and Illustrator (Adobe) and resized when necessary by bicubic interpolation with minimal changes at normal magnifications. Images for the same sets of experiments and controls were adjusted using the same settings to fill in the signal range over full output grayscale over the entire image.

### Immunoprecipitation

HeLa cells in 10-cm Petri dishes were transfected with 10 μg of Lmo1656-FLAG and 7 μg of SNX6-GFP or 7 μg of GFP (amount per dish) using FuGENE (Promega) according to the manufacturer's instruction. Twenty-four hours after transfection, the cells were washed twice in PBS and lysed for 30 min with 1 ml of lysis buffer/10-cm dish (20 mm Tris, pH 8.0, 150 mm NaCl, and 0.5% Triton X-100) supplemented with protease and phosphatase inhibitors. Lysis and all subsequent steps were performed at 4 °C. The lysate was clarified (13,000 × *g*, 10 min), and the protein concentration of the supernatant was determined by Bradford assay (Pierce). 1 mg of lysate was incubated overnight with 30 μl of anti-FLAG-agarose beads (Sigma). Immune complexes were retrieved by centrifugation (500 × *g*, 5 min). After four washes with lysis buffer, bound protein was eluted from the beads by boiling for 10 min in 30 μl of Laemmli buffer. The eluate was analyzed by gradient SDS-PAGE (Bio-Rad) and subjected to Western blotting via wet transfer to 0.45-μm nitrocellulose membrane (Millipore).

## Author contributions

D. J. D., A. P., L. R., and P. C. conceptualization; D. J. D., A. P., and L. R. data curation; D. J. D., A. P., and L. R. formal analysis; D. J. D. and P. C. funding acquisition; D. J. D., A. P., and L. R. investigation; D. J. D., A. P., and L. R. visualization; D. J. D., L. R., M.-A. N., and P. C. methodology; D. J. D. and L. R. writing-original draft; D. J. D. and P. C. project administration; D. J. D., A. P., L. R., and P. C. writing-review and editing; A. P. and P. C. resources; A. P. and P. C. supervision; A. P. and L. R. validation; M.-A. N. animal model work.

## Supplementary Material

Supporting Information

## Footnotes

5 The abbreviations used are:

*Lm*: *L. monocytogenes*
SNX: sorting nexin
LLO: listeriolysin O
SUMO: small ubiquitin-like modifier
InlC: internalin C
BAR: Bin/amphiphysin/Rvs
EF: elongation factor
BHI: brain–heart infusion growth medium
hpi: h postinfection
Cad: cadherin
SCV: *Salmonella*-containing vacuole
CI-MPR: cation-independent mannose 6-phosphate receptor
m.o.i.: multiplicity of infection.

## Note added in proof

In the version of this article that was published as a Paper in Press on April 17, 2018, Tables 1 and 2 were inadvertently omitted. This error has now been corrected.
