# Supplementary material for: Lmo1656 is a secreted virulence factor of Listeria monocytogenes that interacts with the sorting nexin 6–BAR complex
Source: J Biol Chem. 2018 Apr 17;293(24):9265–76. doi: 10.1074/jbc.RA117.000365 (PMC6005434; doi:10.1074/jbc.RA117.000365)
Supplement: Supporting Information [file supp_293_24_9265__index.html]

­Lmo1656 is a secreted virulence factor of Listeria monocytogenes that interacts with the sortin nexin 6-BAR complex — Lmo1656 interacts with the SNX6-BAR complex — Lmo1656 is a secreted virulence factor of Listeria monocytogenes that interacts with the sorting nexin 6–BAR complex — Lmo1656 interacts with the SNX–BAR complex — Supporting Information 

# Lmo1656 is a secreted virulence factor of *Listeria monocytogenes* that interacts with the sorting nexin 6–BAR complex

## Supporting Information

- Supporting Information - &#x00AC;Lmo1656 is a secreted virulence factor of Listeria monocytogenes that interacts with the sortin nexin 6-BAR complex
