# Supplementary material for: Lmo1656 is a secreted virulence factor of Listeria monocytogenes that interacts with the sorting nexin 6–BAR complex
Source: J Biol Chem. 2018 Apr 17;293(24):9265–76. doi: 10.1074/jbc.RA117.000365 (PMC6005434; doi:10.1074/jbc.RA117.000365)
Supplement: Supporting Information [file supp_RA117.000365_133033_2_supp_112189_p61klm.pdf]

Lmo1656 is a secreted virulence factor of *Listeria monocytogenes* that interacts with the sortin nexin 6-BAR complex

Daryl J. V. David[1,§†], Alessandro Pagliuso[1,§], Lilliana Radoshevich[1], Marie-Anne Nahori[1], and Pascale Cossart[1,‡*]

List of material included:

Supplemental Figure 1

Supplemental Figure 2

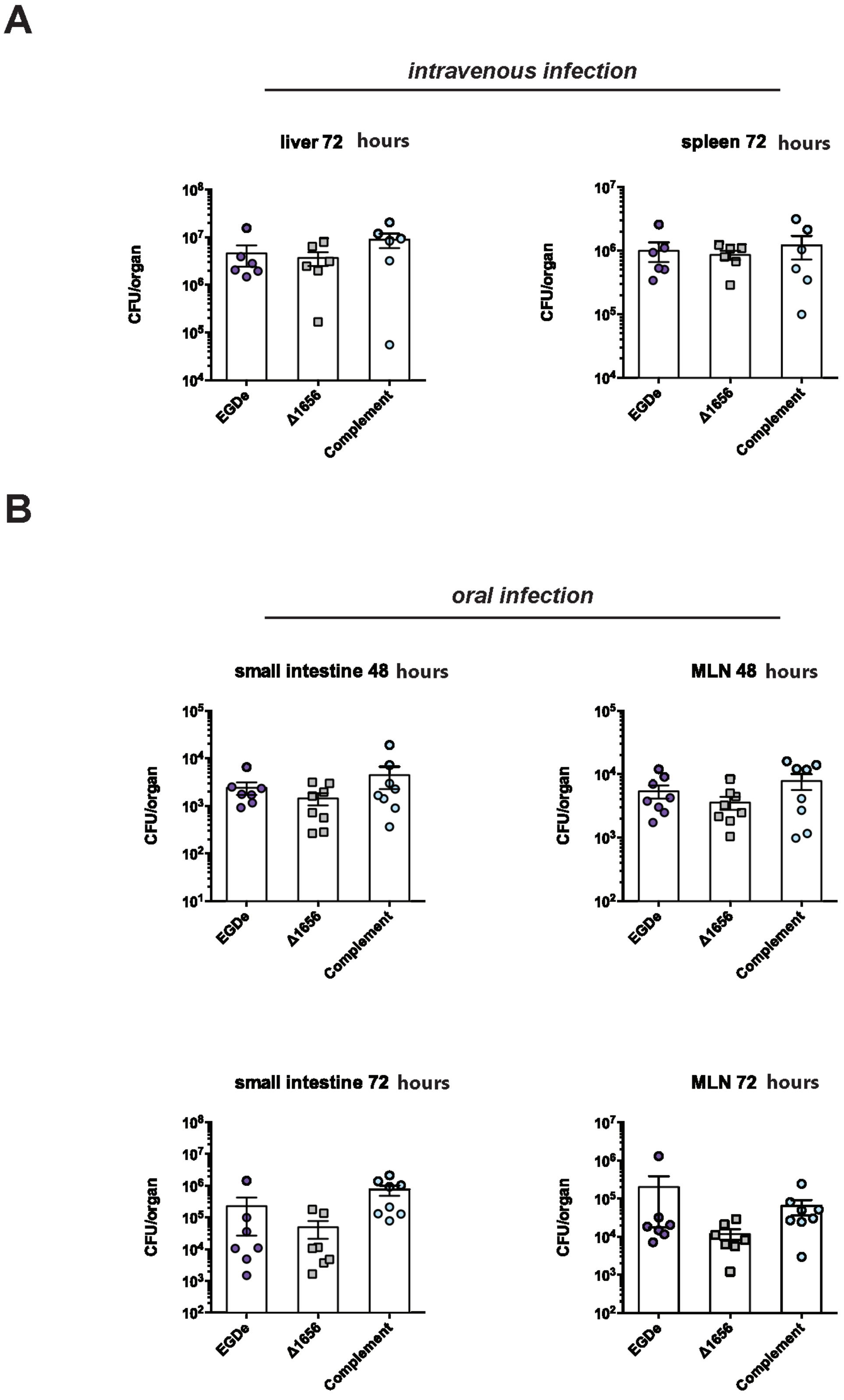

David et al, 2017
Supplemental Figure 1

Supplemental Figure 1: Lmo1656 is a *bona fide* virulence factor of *Listeria monocytogenes*

A. *Lmo1656* does not contribute to virulence in mice infected intravenously. BALB/c mice were infected with either *Lm* ^{WT}, *Lm* $^{\Delta lmo1656}$, or the complemented *Lm* $^{\Delta lmo1656+C}$ intravenously. (n=6 mice per *Lm* genotype). B. *Lmo1656* does not significantly affect bacterial burden in the mesenteric lymph nodes and the intestinal contents of orally-infected mice. These results correspond to the same animals infected in (**Figure 3C,3D**).

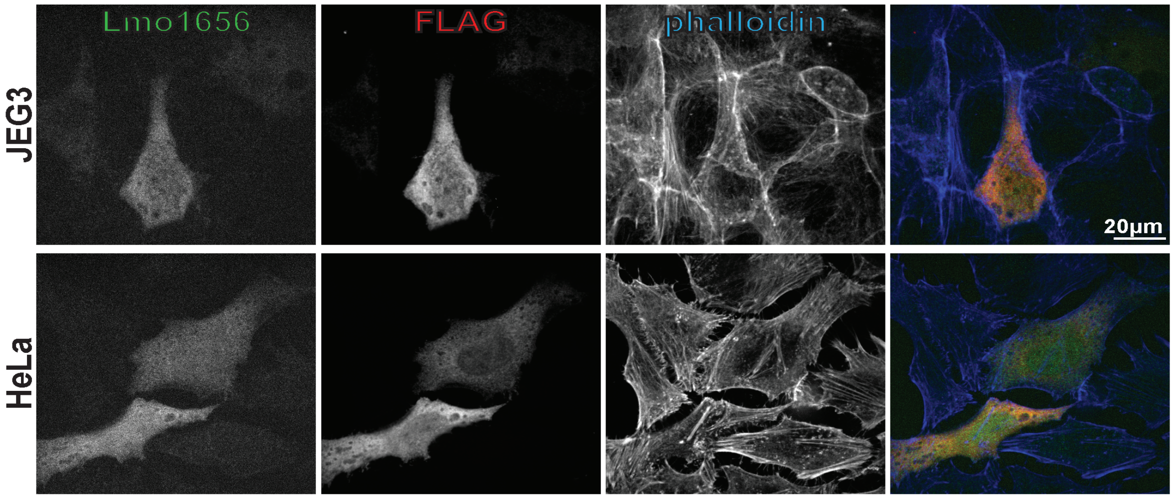

David et al, 2017
Supplemental Figure 2

Supplemental Figure 2: Lmo1656-FLAG shows a diffuse cytoplasmic staining
Cells were transfected with a plasmid encoding Lmo1656. Twenty-four hours post transfection cell were stained with the indicated antibodies.
